# Prediction of Bending, Buckling and Free-Vibration Behaviors of 3D Textile Composite Plates by Using VAM-Based Equivalent Model

**DOI:** 10.3390/ma15010134

**Published:** 2021-12-24

**Authors:** Senbiao Xi, Yifeng Zhong, Zheng Shi, Qingshan Yi

**Affiliations:** 1 School of Civil Engineering, Chongqing University, Chongqing 400045, China; 201816131126@cqu.edu.cn (S.X.); 20191601513@cqu.edu.cn (Z.S.); 20154673@cqu.edu.cn (Q.Y.); 2 Key Laboratory of New Technology for Construction of Cities in Mountain Area, Chongqing University, Chongqing 400045, China

**Keywords:** 3D textile composite, variational asymptotic method, equivalent model, buckling analysis, free-vibration analysis

## Abstract

To solve the microstructure-related complexity of a three-dimensional textile composite, a novel equivalent model was established based on the variational asymptotic method. The constitutive modeling of 3D unit cell within the plate was performed to obtain the equivalent stiffness, which can be inputted into the 2D equivalent model (2D-EPM) to perform the bending, free-vibration and buckling analysis. The correctness and effectiveness of the 2D-EPM was validated by comparing with the results from 3D FE model (3D-FEM) under various conditions. The influence of yarn width and spacing on the equivalent stiffness was also discussed. Finally, the effective performances of 3D textile composite plate and 2D plain-woven laminate with the same thickness and yarn content were compared. The results revealed that the bending, buckling and free-vibration behaviors predicted by 2D-EPM were in good agreement with 3D-FEM, and the local field distributions within the unit cell of 3D textile composite plate were well captured. Compared with the 2D plain-woven laminate, the displacement of 3D textile composite plate was relatively larger under the uniform load, which may due to the fact that the through-the-thickness constrains of the former are only dependent on the binder yarns, while the warp yarns and weft yarns of the latter are intertwined closely.

## 1. Introduction

In recent years, composite structures are more and more widely used as load-bearing structures [1,2,3,4]. Three-dimensional (3D) textile composites is a new type of high performance composites developed from the traditional two-dimensional (2D) textile composites in the 1980s. Compared with 2D textile composites, the warp yarns, weft yarns and binder yarns are interlaced with each other not only in the plane, but also in the thickness direction, which can not only improve the specific strength and specific stiffness of composites, but also have other excellent mechanical properties, such as good impact damage resistance, fatigue resistance, etc. [5]. At the same time, it also overcomes the shortcomings of laminated composite that are easy to delaminate after loading. Another significant benefit of 3D textile composites is the ability to manufacture structural component reforms directly from the yarns.

The application of 3D textile composites in load-bearing structures requires a thorough structural analysis. However, precisely predicting the behaviors of textile composites is difficult due to their complex microstructures. Currently, experimental [6], analytical and numerical methods are used to study the elastic behavior of 3D textile composites.

A variety of analytical models have been used to study the mechanical characteristics of 3D braided composites, including fabric geometry model [7], fiber inclination model [8], three cell model [9], mixed volume averaging technique [10], and Mori-Tanaka theories combined with stiffness averaging method [11]. Yang et al. [12] introduced the “Fiber Inclination Model” to estimate the elastic properties of 3D textile (woven and braided) composites. The unit cell employed for the study was an assemblage of inclined unidirectional laminae. Based on the classical laminate plate theory (CLPT) and iso-stress/strain assumptions, Ishikawa and Chou [13,14] presented analytical methods to estimate the homogenized response of woven fabric composites. Branch et al. [15] presented a 3D tow inclination model for calculating the elastic constants of three-dimensional braided composites. The global constitutive equation of the composite material was derived by applying an iso-strain method to the unit cell and averaging all tow segments and the matrix inside the unit cell. Yan et al. [16] predicted the properties of 3D braided structures using an analytical model called the Fabric Geometry Model (FGM). Using the stiffness volume average method and Tsai-Wu polynomial failure criterion, Jiang et al. [17] presented a theoretical model based on the helix geometry unit cell for prediction of the effective elastic constants and the failure strength of 3D braided composites under uniaxial load. Analytical models are good at estimating the in-plane properties of textile composites, but they are not so good at predicting the shear and out-of-plane properties.

Although utilizing experimental and analytical models to examine the mechanical characteristics of a laminated composite plate is practical and efficient [18], many studies also employed numerical approaches to investigate the 3D textile composites [19,20,21]. Tan et al. [22] developed a mesoscale finite element model (MSFEM) in LS-DYNA to simulate impact damage to three-dimensional braided composite plates based on the assumption that the fiber yarn was made up of cylindrical segments. Dong et al. [23] simulated the micro-stress of 3D braided composites by the method of Asymptotic Expansion Homogenization (AEH) combined with finite-element analysis. Tang and Whitcomb [24] used the full multiscale mechanical model to perform progressive failure evaluations of 2 × 2 braided composites. This approach was also used by Potluri and Manan to investigate the mechanical characteristics of braided composite tubes [25], but the stress and strain distribution of the fiber and matrix cannot be determined.

Berdichevsky [26] recently developed the semi-analytical approach-variational asymptotic technique (VAM) to increase the efficiency of numerical methods and the accuracy of analytical methods. It combines the benefits of asymptotic and variational methods, and takes into account all potential deformations [27,28,29]. The fundamental benefit of adopting VAM for plate analysis is that it can divide the original 3D plate problem into two independent problems using the small parameter of thickness-width ratio, i.e., through-the-thickness analysis and 2D reference plane analysis [30,31]. Then, VAM was expanded by Zhong and Yu to simulate piezoelectric and piezomagnetic laminates [32], multilayer graded magnetoelectroelastic plates [33] and composite cylindrical shells [34].

The VAM model is expanded in this article to present an equivalent plate model to replace the original 3D textile composite plate (3D-TCP) for bending, buckling, and free-vibration analysis. The influences of structural parameters (binder yarn width, warp or weft yarn width) on the equivalent stiffness of 3D-TCP are investigated. Finally, the effective performance of 2D plain-woven laminate (2D-PWL) and 3D-TCP with the same plate thickness and yarn content are compared. To the best of the authors’ knowledge, this technique has never been used to predict the bending, buckling, and free-vibration behaviors of 3D-TCP.

## 2. Variational Asymptotic Equivalent Model of 3D-TCP

**Figure 1:**
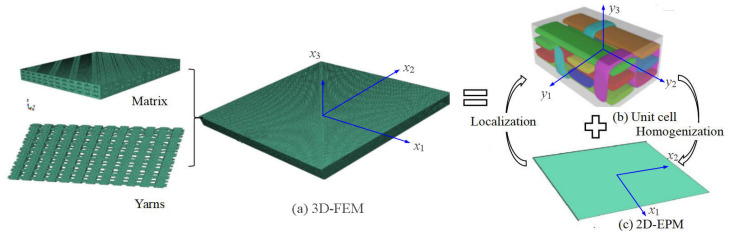
Decomposition diagram of 3D textile composite plate: (**a**) 3D-FEM; (**b**) Unit cell; (**c**) 2D-EPM.

As illustrated in Figure 1a, the macro-coordinates $x_{i}\left(i=1,2,3\right)$ may be used to describe any point in the 3D-TCP, where $x_{\alpha }\left(\alpha =1,2\right)$ are the in-plane coordinates and $x_{3}$ is the normal coordinate. The 3D finite element model of 3D-TCP may be divided into the 3D unit cell and 2D equivalent plat model (2D-EPM) according to the VAM. It’s worth mentioning that the dimensions of the unit cell should be substantially smaller than macro-structure dimensions. As illustrated in Figure 1c, the field variables of 2D-EPM are represented as functions of $x_{1}$ and $x_{2}$, whereas $x_{3}$ is disappeared.

To characterize the quick change of in-plane material characteristics, the micro-coordinates $y_{i}=x_{i}/\zeta$ (ζ is a small parameter) are introduced. To obtain the equivalent model of 3D-TCP by using the variational asymptotic method, the 3D displacement field of the original 3D-TCP needs to be represented by 2D plate variables, such as

(1) $$\begin{array}{c} u_{1}\left(x_{\alpha };y_{i}\right)=\underline{{\bar{u}}_{1}\left(x_{\alpha }\right)-\zeta y_{3}{\bar{u}}_{3,1}\left(x_{\alpha }\right)}+\zeta w_{1}\left(x_{\alpha };y_{i}\right)\\ u_{2}\left(x_{\alpha };y_{i}\right)=\underline{{\bar{u}}_{2}\left(x_{\alpha }\right)-\zeta y_{3}{\bar{u}}_{3,2}\left(x_{\alpha }\right)}+\zeta w_{2}\left(x_{\alpha };y_{i}\right)\\ u_{3}\left(x_{\alpha };y_{i}\right)=\underline{{\bar{u}}_{3}\left(x_{\alpha }\right)}+\zeta w_{3}\left(x_{\alpha };y_{i}\right) \end{array}$$

where the displacements of the 3D-FEM and 2D-EPM are represented by $u_{i}$ and ${\bar{u}}_{i}$, respectively; $w_{i}$ are the fluctuation functions to be solved. The underline terms should satisfy the following constraints:

(2) $$h{\bar{u}}_{\alpha }\left(x_{\alpha }\right)=\langle u_{\alpha }\rangle +\langle \zeta y_{3}\rangle {\bar{u}}_{3,\alpha },\;h{\bar{u}}_{3}\left(x_{\alpha }\right)=\langle u_{3}\rangle$$

where 〈·〉 denotes the volume integral of a unit cell.

The fluctuation functions in Equation (3) are constrained as

(3) $$\langle \zeta w_{i}\rangle =0$$

The strain field can be expressed according to 3D linear elasticity theory, such as

(4) $$\varepsilon_{ij}=\frac{1}{2}\left(\frac{\partial u_{i}}{\partial x_{j}}+\frac{\partial u_{j}}{\partial x_{i}}\right)$$

The 3D strain field can be obtained by substituting Equation (1) into Equation (4) and ignoring higher-order terms according to VAM,

(5) $$\begin{array}{c} \varepsilon_{11}=\gamma_{11}+\zeta y_{3}\kappa_{11}+w_{1,1}\\ 2\varepsilon_{12}=2\gamma_{12}+2\zeta y_{3}\kappa_{12}+w_{1,2}+w_{2,1}\\ \varepsilon_{22}=\gamma_{22}+\zeta y_{3}\kappa_{22}+w_{2,2}\\ 2\varepsilon_{13}=w_{1,3}+w_{3,1}\\ 2\varepsilon_{23}=w_{2,3}+w_{3,2}\\ \varepsilon_{33}=w_{3,3} \end{array}$$

where $\gamma_{\alpha \beta }$ and $\kappa_{\alpha \beta }$ are the in-plane strains and bending curvatures of 2D-EPM, respectively, and may be defined as

(6) $$\gamma_{\alpha \beta }\left(x_{1},x_{2}\right)=\frac{1}{2}\left({\bar{u}}_{\alpha ,\beta }+{\bar{u}}_{\beta ,\alpha }\right),\;\kappa_{\alpha \beta }\left(x_{1},x_{2}\right)=-{\bar{u}}_{3,\alpha \beta }$$

We can define the three-dimensional strain field in matrix form to make derivation easier, such as

(7) $$\begin{array}{c} \varepsilon_{e}={\left[\varepsilon_{11}\;\varepsilon_{22}\;2\varepsilon_{12}\right]}^{T}=\gamma +x_{3}\kappa +I_{\alpha }w_{||,\alpha }\\ 2\varepsilon_{s}={\left[2\varepsilon_{13}\;2\varepsilon_{23}\right]}^{T}=w_{||,3}+e_{\alpha }w_{3,\alpha }\\ \varepsilon_{t}=\varepsilon_{33}=w_{3,3} \end{array}$$

where ${\left(\right)}_{\left|\right|}={\left[{\left(\right)}_{1}\;{\left(\right)}_{2}\right]}^{T}$, $\gamma ={\left[\begin{array}{ccc} \gamma_{11} & 2\gamma_{12} & \gamma_{22} \end{array}\right]}^{T}$, $\kappa ={\left[\kappa_{11}\;\kappa_{12}+\kappa_{21}\;\kappa_{22}\right]}^{T}$, and

(8) $$I_{1}=\left[\begin{array}{cc} 1 & 0\\ 0 & 1\\ 0 & 0 \end{array}\right],I_{2}=\left[\begin{array}{cc} 0 & 0\\ 1 & 0\\ 0 & 1 \end{array}\right],e_{1}=\left\{\begin{array}{c} 1\\ 0 \end{array}\right\},e_{2}=\left\{\begin{array}{c} 0\\ 1 \end{array}\right\}$$

The strain energy of the 3D-TCP may be expressed briefly as

(9) $$U=\frac{1}{2}\langle \varepsilon^{T}D\varepsilon \rangle =\frac{1}{2}\langle {\left\{\begin{array}{c} \varepsilon_{e}\\ 2\varepsilon_{s}\\ \varepsilon_{t} \end{array}\right\}}^{T}\left[\begin{array}{ccc} D_{e} & D_{es} & D_{et}\\ D_{es}^{T} & D_{s} & D_{st}\\ D_{et}^{T} & D_{st}^{T} & D_{t} \end{array}\right]\left\{\begin{array}{c} \varepsilon_{e}\\ 2\varepsilon_{s}\\ \varepsilon_{t} \end{array}\right\}\rangle$$

where $D_{e},D_{es},D_{et},D_{s},D_{st}$ and $D_{t}$ are the corresponding sub-matrices of a 3D 6×6 material matrix.

The virtual work done by the applied load may be stated as

(10) $$\delta {\bar{W}}_{3D}=\delta {\bar{W}}_{2D}+\delta {\bar{W}}^{*}$$

where $\delta {\bar{W}}_{2D}$ and $\delta {\bar{W}}^{*}$ denote, respectively, the virtual work independent and dependent of the fluctuation function, and

(11) $$\delta {\bar{W}}_{2D}=\langle p_{i}\delta v_{i}+q_{a}\delta v_{3,a}\rangle ,\delta {\bar{W}}^{*}=\langle \langle f_{i}\delta w_{i}\rangle +\tau_{i}\delta w_{i}^{+}+\beta_{i}\delta w_{i}^{-}\rangle$$

where ${\left(\cdot \right)}^{+}$ and ${\left(\cdot \right)}^{-}$ represent the items acting on the top and bottom of the plate, respectively; $\tau_{i}$ and $\beta_{i}$ are the traction forces on the top and bottom surface of the plate, respectively; $f_{i}$ are the body forces; $p_{i}=\langle f_{i}\rangle +a_{i}+\beta_{i},q_{a}=h/2\left(\beta_{a}-a_{a}\right)-\langle x_{3}f_{a}\rangle$.

The variation of the total potential energy may be written as

(12) $$\delta \Pi =\delta U-\delta W^{*}=\frac{1}{2}\delta \langle \varepsilon^{T}D\varepsilon \rangle -\langle \langle f_{i}\delta w_{i}\rangle +\tau_{i}\delta w_{i}^{+}+\beta_{i}\delta w_{i}^{-}\rangle$$

where only the unknown fluctuation function $w_{i}$ is changeable.

### 2.1. Dimensional Reduction Analysis of 3D-TCP

The zeroth-order fluctuation function may be solved by minimizing the zeroth-order approximation strain energy under the constraint of Equation (9) as

(13) $$\delta U_{0}=0$$

where

(14) $$2U_{0}=\langle \begin{array}{c} {\left(\gamma +x_{3}\kappa \right)}^{T}D_{e}\left(\gamma +x_{3}\kappa \right)+w_{||,3}^{T}D_{s}w_{||,3}+w_{3,3}^{T}D_{t}w_{3,3}\\ +2{\left(\gamma +x_{3}\kappa \right)}^{T}\left(D_{es}w_{||,3}+D_{et}w_{3,3}\right)+2w_{||,3}^{T}D_{st}w_{3,3} \end{array}\rangle$$

The Lagrange multipliers $\lambda_{i}$ are used to impose the constraint on the fluctuation function as

(15) $$\delta \left(\Pi +\lambda_{i}\langle w_{i}\rangle \right)=0$$

The zeroth-order approximate variational expression can be obtained as

(16) $$\langle \begin{array}{c} \left[{\left(\gamma +x_{3}\kappa \right)}^{T}D_{es}+w_{||,3}^{T}D_{s}+w_{3,3}^{T}D_{st}^{T}\right]\delta w_{||,3}\\ +\lambda_{i}\delta w_{i}+\left[{\left(\gamma +x_{3}\kappa \right)}^{T}D_{et}+w_{||,3}^{T}D_{st}+w_{3,3}^{T}D_{t}\right]\delta w_{3,3} \end{array}\rangle =0$$

By partly integrating Equation (16), the relevant Euler-Lagrange equation may be obtained as

(17) $$\begin{array}{c} {\left[{\left(\gamma +x_{3}\kappa \right)}^{T}D_{es}+{\left(w_{||,3}\right)}^{T}D_{s}+w_{3,3}D_{st}\right]}_{,3}=\lambda_{\left|\right|}\\ {\left[{\left(\gamma +x_{3}\kappa \right)}^{T}D_{et}+{\left(w_{||,3}\right)}^{T}D_{st}+w_{3,3}D_{t}\right]}_{,3}=\lambda_{3} \end{array}$$

where $\lambda_{\left|\right|}={\left[\lambda_{1}\;\lambda_{2}\right]}^{T}$.

According to the free surface condition, the expressions in square brackets of Equation (17) should be zero at the top and bottom of the plate, such as

(18) $$\begin{array}{c} {\left[{\left(\gamma +x_{3}\kappa \right)}^{T}D_{es}+w_{||,3}^{T}D_{s}+w_{3,3}D_{st}^{T}\right]}^{+/-}=0\\ {\left[{\left(\gamma +x_{3}\kappa \right)}^{T}D_{et}+w_{||,3}^{T}D_{st}+w_{3,3}D_{t}\right]}^{+/-}=0 \end{array}$$

where the superscript “+/−” denotes the items at the top and bottom of the plate.

$w_{\left|\right|}$ and $w_{3}$ can be solved by putting Equation (18) into Equation (17), such as

(19) $$w_{\left|\right|}={\langle -\left(\gamma +x_{3}\kappa \right){\bar{D}}_{es}{\left(D_{s}\right)}^{-1}\rangle }^{T},w_{3}=\langle -\left(\gamma +x_{3}\kappa \right){\bar{D}}_{et}{\left(D_{t}\right)}^{-1}\rangle$$

where

(20) $$\begin{array}{c} {\bar{D}}_{es}=D_{es}-{\bar{D}}_{et}{\left(D_{st}\right)}^{T}{\left({\bar{D}}_{t}\right)}^{-1},\;{\bar{D}}_{et}=D_{et}-D_{es}{\left(D_{s}\right)}^{-1}D_{st},\\ {\bar{D}}_{t}=D_{t}-{\left(D_{st}\right)}^{T}{\left(D_{s}\right)}^{-1}D_{st} \end{array}$$

Substituting Equation (20) into Equation (14), we obtain

(21) $$\begin{array}{c} U_{2D}=\frac{1}{2}\langle {\left(\gamma +x_{3}\kappa \right)}^{T}{\bar{D}}_{e}\left(\gamma +x_{3}\kappa \right)\rangle =\frac{1}{2}{\left\{\begin{array}{c} \gamma \\ \kappa \end{array}\right\}}^{T}\left[\begin{array}{cc} A & B\\ B^{T} & D \end{array}\right]\left\{\begin{array}{c} \gamma \\ \kappa \end{array}\right\}\\ =\frac{1}{2}{\left\{\begin{array}{c} \gamma_{11}\\ \gamma_{22}\\ 2\gamma_{12}\\ \kappa_{11}\\ \kappa_{22}\\ 2\kappa_{12} \end{array}\right\}}^{T}\left[\begin{array}{cccccc} A_{11} & A_{12} & A_{16} & B_{11} & B_{12} & B_{16}\\ A_{12} & A_{22} & A_{26} & B_{12} & B_{22} & B_{26}\\ A_{16} & A_{26} & A_{66} & B_{16} & B_{26} & B_{66}\\ B_{11} & B_{12} & B_{16} & D_{11} & D_{12} & D_{16}\\ B_{12} & B_{22} & B_{26} & D_{12} & D_{22} & D_{26}\\ B_{16} & B_{26} & B_{66} & D_{16} & D_{26} & D_{66} \end{array}\right]\left\{\begin{array}{c} \gamma_{11}\\ \gamma_{22}\\ 2\gamma_{12}\\ \kappa_{11}\\ \kappa_{22}\\ 2\kappa_{12} \end{array}\right\} \end{array}$$

where A, D and B are tensile, bending, and coupling stiffness sub-matrices, respectively, and can be expressed as

(22) $$\begin{array}{c} A=\langle \langle {\bar{D}}_{e}\rangle \rangle ,B=\langle \langle x_{3}{\bar{D}}_{e}\rangle \rangle ,D=\langle \langle x_{3}^{2}{\bar{D}}_{e}\rangle \rangle ,\\ {\bar{D}}_{e}=D_{e}-{\bar{D}}_{es}D_{s}^{-1}D_{es}^{T}-{\bar{D}}_{et}D_{et}^{T}/{\bar{D}}_{t} \end{array}$$

The stiffness matrix provides the essential information of 3D-TCP and may be easily utilized in the shell elements in a finite element software to perform macroscopic plate analysis. Because it only concerns the 2D field variables in terms of the macro-coordinates $x_{1}$ and $x_{2}$, the macroscopic behavior of the plate is governed by the the strain energy in Equation (21). As a result, the 2D-EPM may be used to represent the original 3D-TCP in the global analysis, and can be solved using the linear analysis solver in a finite element soft package like ABAQUS/Standard.

### 2.2. Local Field Analysis

A well-established equivalent model may be applied not only to global analysis, but also to local field analysis. To improve the equivalent model, the local field recovery relations should be given.

Equation (3) may be used to recover the local 3D displacement field, such as

(23) $$u_{i}={\bar{u}}_{i}+\left[\begin{array}{ccc} {\bar{u}}_{1,1} & {\bar{u}}_{1,2} & {\bar{u}}_{1,3}\\ {\bar{u}}_{2,1} & {\bar{u}}_{2,2} & {\bar{u}}_{2,3}\\ {\bar{u}}_{3,1} & {\bar{u}}_{3,2} & {\bar{u}}_{3,3} \end{array}\right]\left\{\begin{array}{c} y_{1}\\ y_{2}\\ y_{3} \end{array}\right\}+\zeta w_{i}$$

The local strain field can be recovered as

(24) $$\varepsilon_{e}^{0}=\gamma +x_{3}\kappa ,\;2\varepsilon_{s}^{0}=-w_{||,3},\;\varepsilon_{t}^{0}=w_{3,3}$$

The Hooke’s law may be used to recover the local stress field as

(25) $$\sigma =\bar{D}\varepsilon$$

## 3. Validation Example

**Figure 2:**
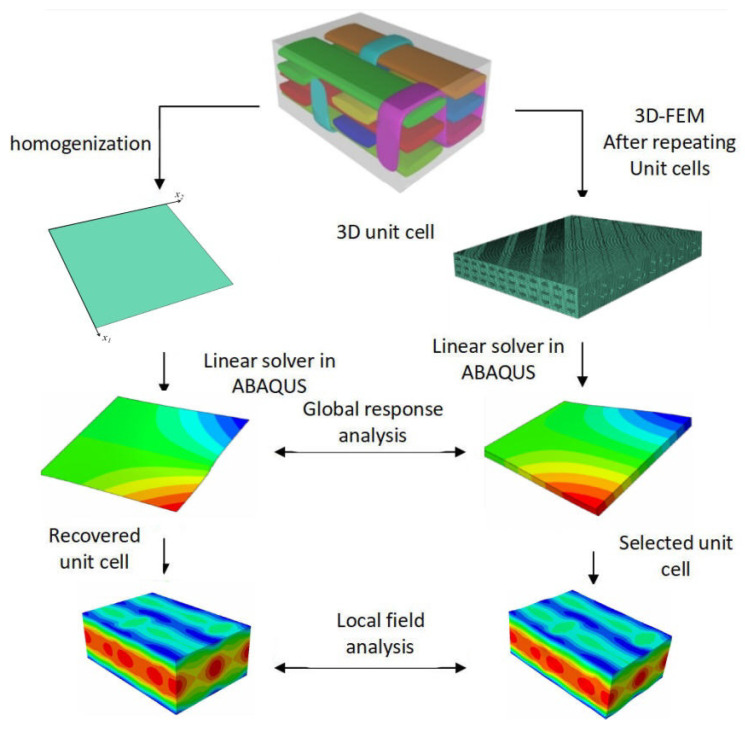
Comparative analysis of 2D-EPM and 3D-FEM.

In this part, numerical examples of bending, buckling, and free vibration of 3D-TCP under various conditions are utilized to validate the accuracy and efficiency of 2D-EPM. The comparative analysis is depicted in Figure 2. The relative error between 2D-EPM and 3D-FEM is calculated as

(26) $$Error=\frac{|2D-\mathrm{EPM}\;\mathrm{results}-3D-\mathrm{FEM}\;\mathrm{results}|}{3D-\mathrm{FEM}\;\mathrm{results}}\times 100\%.$$

**Figure 3:**
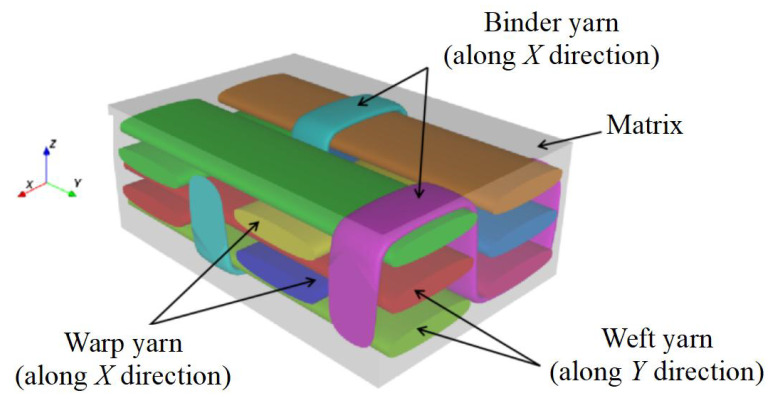
The unit cell of 3D textile composite plate.

**Figure 4:**
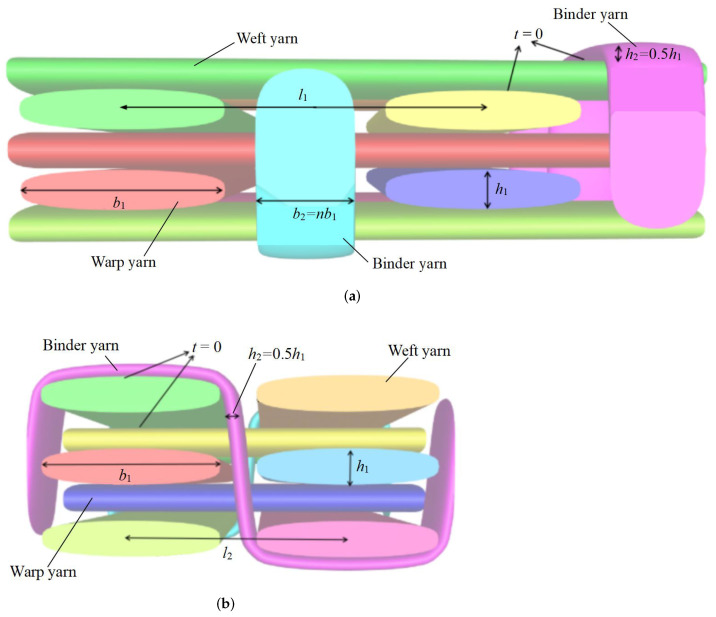
Structural parameters of unit cell within the 3D-TCP. (**a**) *Y* direction; (**b**) *X* direction.

The microstructure of 3D textile composite plate as shown in Figure 3 is very complex, including three layers with two weft yarns in each layer, two layers with two warp yarns in each layer and two binder yarns. The geometry of unit cell is determined by several parameters as shown in Figure 4: (1) the interval *t* between layers along the *Z* direction; (2) the interval length $l_{1}$ between the weft yarns along the *Y* direction; (3) the interval length $l_{2}$ between the warp yarns along the *X* direction, where $l_{2}$ = $nl_{1}$ (n<1); (4) the warp or weft yarn width $b_{1}$ and the thickness $h_{1}$; and (5) the binder yarn width $b_{2}=nb_{1}$ and the thickness $h_{2}=0.5h_{1}$. The *X*, *Y* and *Z* direction of the unit cell are the same as $y_{1}$, $y_{2}$ and $y_{3}$ in the theoretical derivation, respectively.

**Figure 5:**
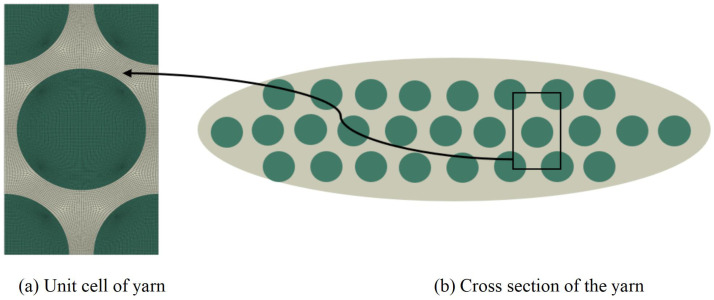
Unit cell model of the yarn.

In this section, these parameters are set as: $b_{1}$ = 0.8, $h_{1}$ = 0.2 mm, $l_{1}$ = 1.2 mm, $b_{2}$ = 0.4 mm, $h_{2}$ = 0.1 mm, $l_{2}$ = 1.0 mm, and *t* = 0 mm. The dimensions of unit cell are 3.6mm×2.4mm×1.32mm. The constituents in the composite material are carbon fiber (T-300) and epoxy resin 3601, and their properties are obtained from ref. [35], as shown in Table 1. The yarn has elliptical cross-section as shown in Figure 5b, and usually modeled as unidirectional composites with hexagonal pack as shown in Figure 5a. The variational asymptotic homogenization method is used to obtain the equivalent engineering constants of the yarn with 64% fiber volume fraction, as shown in Table 2. The geometry model of unit cell within the 3D-TCP is generated by open source TexGen software. The yarns and matrix, as well as the yarns themselves, are linked by common nodes, indicating that different parts are perfectly connected. The equivalent stiffness matrix of 3D-TCP obtained by the present model is provided in Table 3.

The unit cell is repeated 12 times in the *X* direction and 8 times along the *Y* direction to construct the 3D-FEM of a 3D textile composite plate. The dimensions of this plate are 28.8 mm long, 28.8 mm wide, and 1.32 mm thick, respectively. After the mesh convergence study, a total of 10,609 shell elements (S4R) and 288,000 solid elements (C3D20) are used in 2D-EPM and 3D-FEM, respectively.

### 3.1. Bending Analysis

**Figure 6:**
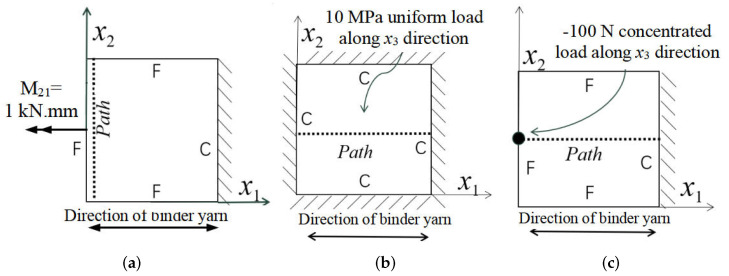
Combinations of boundary and load conditions for bending analysis. (**a**) Case 1; (**b**) Case 2; and (**c**) Case 3.

Three combinations of boundary and load conditions in Figure 6 are used in bending analysis, in which C represents clamped boundary, F for free boundary, and Path represents the comparative analysis path. Table 4 shows the bending behaviors predicted by 3D-FEM and 2D-EPM under various conditions.

**Figure 7:**
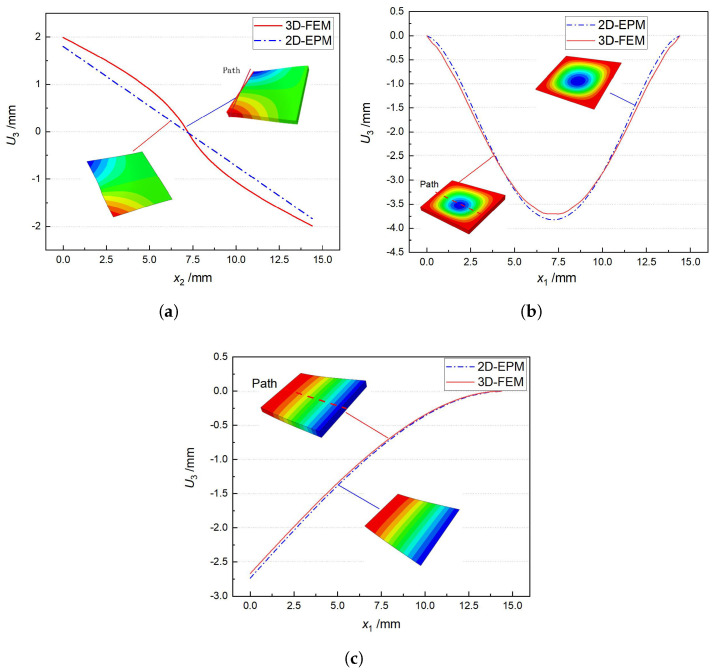
Comparison of displacements along analysis paths under various boundary and load conditions. (**a**) Case 1; (**b**) Case 2; and (**c**) Case 3.

Table 4 shows that the displacement clouds of $U_{3}$ predicted by the two models are consistent, and the maximum error is 7.70% in Case 1, which may due to the fact that the shear strain is negligible in Case 2 and Case 3, but relatively large in Case 1. To more clearly illustrate the details of the displacement distributions (especially out-of-plane distributions), the displacement of $U_{3}$ along the analysis path are compared in Figure 7. It can be observed that the displacement error between 3D-FEM and 2D-EPM is relatively very small even for 3D-TCP with complex microstructures, and the displacement curve of 3D-FEM is smoother than that of 2D-EPM. The main reason is that there is only one node in $x_{3}$ direction of 2D-EPM under bending load, which can not smoothly simulate the continuous deformation along the thickness direction.

### 3.2. Local Field Recovery

**Figure 8:**
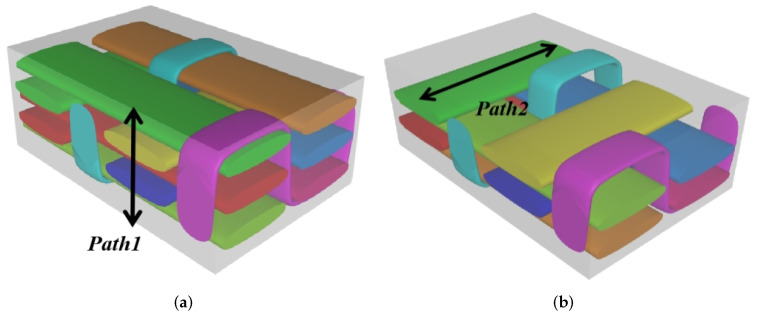
Path selection in local field recovery analysis. (**a**) Path 1 along the *Z* direction; and (**b**) Path 2 along the *Y* direction.

The internal structure of 3D-TCP is complex, and the warp yarns, weft yarns and binder yarns are intertwined with each other. The study of the local stress, strain and displacement distributions is of significance to the failure analysis of textile structures. In this section, the local fields within the unit cell at the center of the plate are recovered under the conditions in Case 2. Two paths (as shown in Figure 8) are selected to analyze the local stress, strain and displacement distribution.

**Figure 9:**
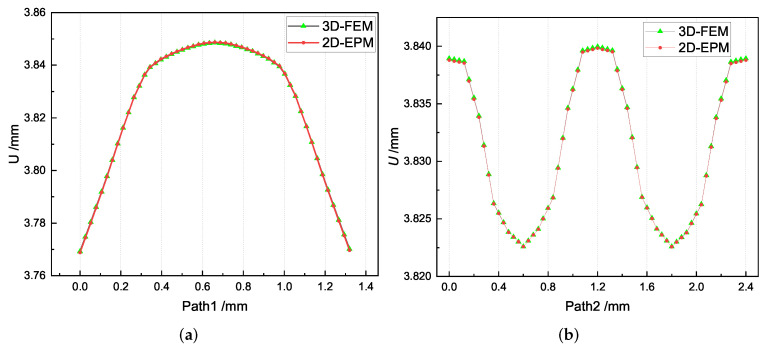
Comparison of local displacement curves along Path 1 and Path 2 of the unit cell predicted by two models under the conditions in Case 2. (**a**) *U* along Path 1; (**b**) *U* along Path 2.

The local displacement distributions within the recovered unit cell from 2D-EPM are similar with those in the selected unit cell from 3D-FEM, as shown in Table 5, with a maximum error of 1.18%. Figure 9a shows that the maximum displacement of *U* is located in the middle of Path 1, where the displacements of weft yarns are greater than those of matrix. Figure 9b shows that the curve of *U* along Path 2 is divided into two segments with a length of 1.2 mm (the interval length between the weft yarns). The larger value of *U* is located in the suspended area between the weft yarns, while the smaller value of *U* is located at 0.5 mm and 1.7 mm of Path 2, which belongs to the superimposed area of the warp yarns and weft yarns. The local stress distributions within the recovered unit cell from 2D-EPM are similar with those in the selected unit cell from 3D-FEM, as shown in Table 6, and the maximum error is is 5.17% in $\sigma_{22}$, indicating that the recovered local stress fields from 2D-EPM are accurate.

**Figure 10:**
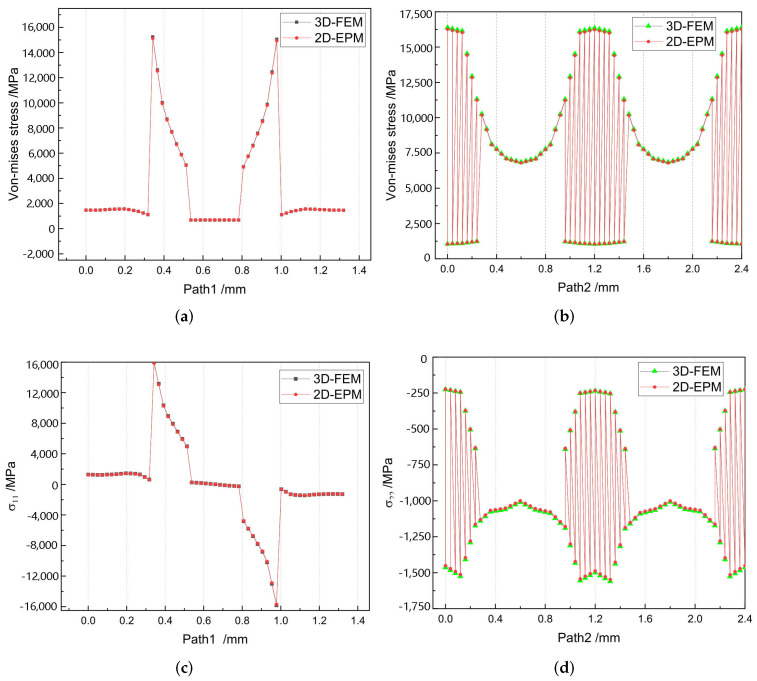
Comparison of local stress distributions along Path 1 and Path 2 of the unit cell predicted by two models under the conditions in Case 2. (**a**) Von mises stress along Path 1; (**b**) Von mises stress along Path 2; (**c**) $\sigma_{11}$ along Path 1; and (**d**) $\sigma_{22}$ along Path 2.

Figure 10a,c show that the curves of von Mises stress and $\sigma_{11}$ along Path 1 are divided into five segments, representing two layers of warp yarns and three layers of matrix, respectively. The local stresses are distributed unevenly within the unit cell of 3D-TCP. The local stress in the matrix is relatively small, while the local stress in the warp yarns fluctuates greatly, indicating that the yarns along the thickness direction are main bearing components. Figure 10b,d show that the curves of von Mises stress and $\sigma_{22}$ along Path 2 are also divided into five sections. The stress at the superimposed area of warp yarns and weft yarns (0.25–0.95 mm, 1.45–2.15 mm) is relatively small, while the stress in the suspended area of the weft yarns fluctuates greatly, which indicates that it is easy to be damaged under the loading.

**Figure 11:**
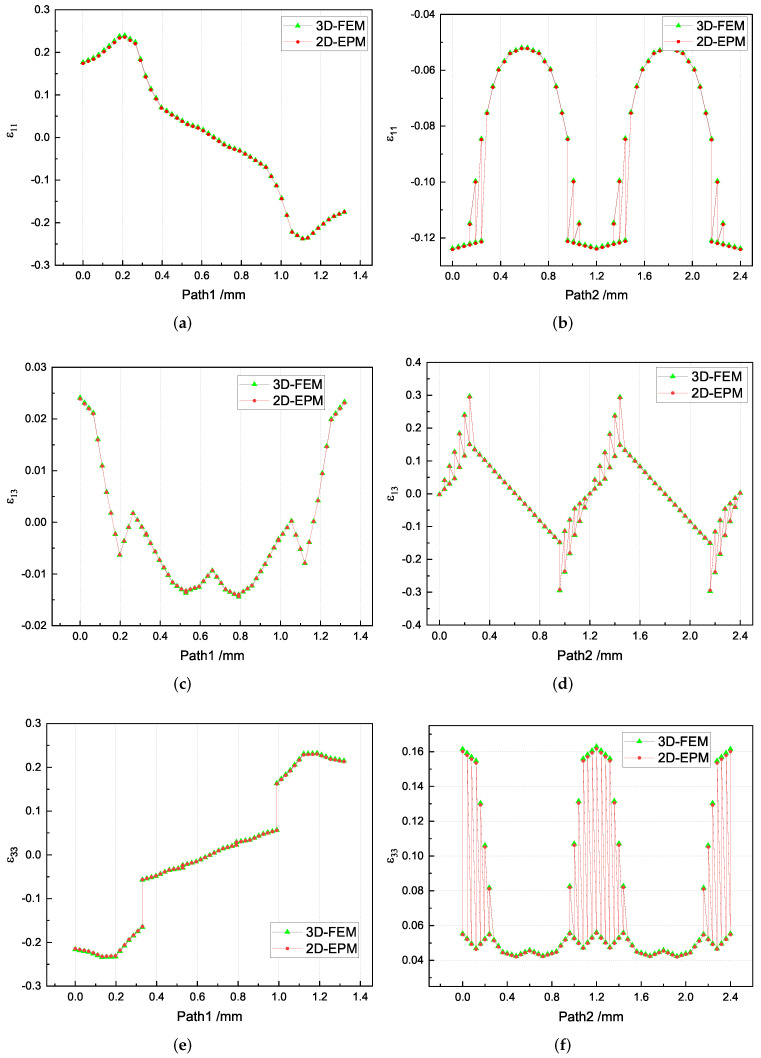
Comparison of local strain curves along Path 1 and Path 2 of the unit cell predicted by two models under the conditions in Case 2. (**a**) $\varepsilon_{11}$ along Path 1; (**b**) $\varepsilon_{11}$ along Path 2; (**c**) $\varepsilon_{13}$ along Path 1; (**d**) $\varepsilon_{13}$ along Path 2; (**e**) $\varepsilon_{33}$ along Path 1; and (**f**) $\varepsilon_{33}$ along Path 2.

Table 7 shows that the local strain distributions are consistent with those of local stress distributions, and the error of local strain between recovered unit cell and selected unit cell is less than 1%. Figure 11 shows the local strain distributions along Path 1 and Path 2 of the unit cell predicted by two models under the conditions in Case 2. It can be observed that the local strain distributions within the unit cell of 3D-TCP are non-homogeneous. The strain curves are divided into several segments due to the interpenetration of warp yarn, weft yarn and matrix.

### 3.3. Global Buckling Analysis

**Figure 12:**
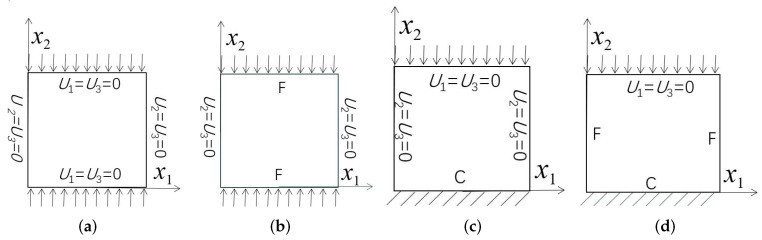
Boundary and load conditions used in bucking analysis. (**a**) Case 4; (**b**) Case 5; (**c**) Case 6; and (**d**) Case 7.

In this section, the global buckling of 3D-TCP under different conditions shown in Figure 12 is analyzed. The opposite sides of the 2D-EPM are subjected to a linear load of 1 N/mm, whereas the opposite sides of the 3D-FEM are subjected to a uniform stress of 1/0.22 = 4.5455 MPa.

Table 8 lists the first six buckling modes and loads of 3D-TCP predicted by the two models under the conditions in Case 6. The first six buckling modes predicted by 3D-FEM and 2D-EPM are consistent, and the maximum error of buckling critical load in each buckling mode is only 2%. The calculation time of 2D-EPM in buckling analysis is about 18 times faster than 3D-FEM, verifying the effectiveness of 2D-EPM in global buckling analysis of 3D-TCP.

Table 9 lists the first buckling modes and critical loads predicted by the two models under the conditions in Case 4, Case 5 and Case 7. The first buckling modes predicted by the two models are nearly identical, and the maximum error of the buckling load is only 2.69%, which verifies the accuracy of 2D-EPM in buckling analysis of 3D-TCP under different conditions.

### 3.4. Free-Vibration Analysis

Table 10 and Table 11 show the first three vibration modes and natural frequencies of 3D-TCP under the boundary conditions in Cases 4 and 7. It is clear that the vibration modes predicted by 2D-EPM agree with those predicted by 3D-FEM. For example, the first, second buckling modes, respectively, have one and two half-waves along the $x_{1}$ axis, and the third buckling mode has two half-waves along the $x_{2}$ axis under the boundary condition in Case 4. The maximum natural frequency error is 8.67%, indicating 2D-EPM has high accuracy in free-vibration analysis of 3D-TCP.

## 4. Influence of Structural Parameters on Equivalent Stiffness

The structure of 3D-TCP is complex and has many parameters (see Figure 3). In Section 3.2, we can see that the local stress and strain in binder yarns are relatively greater, indicating the binder yarns plays a very important role in preventing interlayer separation in 3D-TCP. Therefore, it is very important to study the influence of binder yarn width on the equivalent stiffness, which can also provide guidance for the design of 3D-TCP. Secondly, the influence of warp (weft) yarn width on the equivalent stiffness are also investigated. Table 12 lists the structural parameters of 3D-TCP used in the parameter analysis.

**Figure 13:**
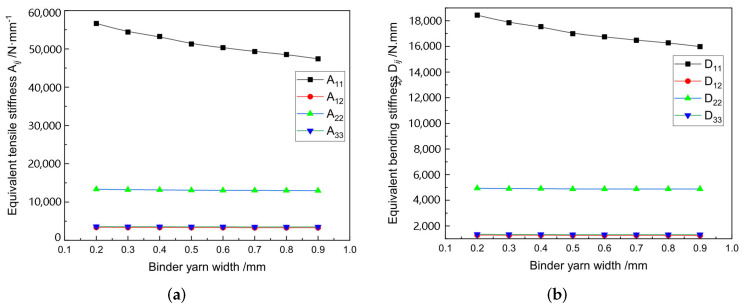
Influence of the binder yarn width on equivalent stiffness of 3D-TCP. (**a**) $A_{ij}$; (**b**) $D_{ij}$.

**Figure 14:**
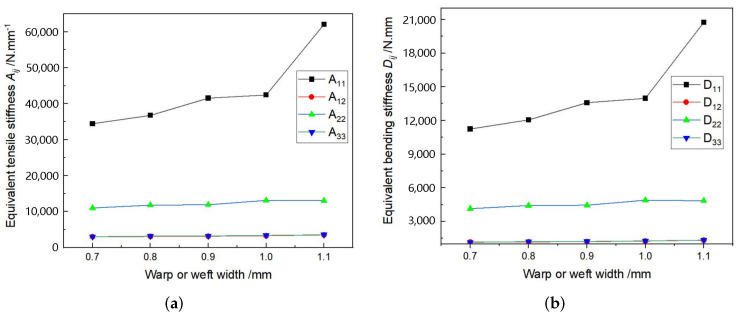
Influence of the warp (weft) yarn width on equivalent stiffness of 3D-TCP. (**a**) $A_{ij}$; (**b**) $D_{ij}$.

Figure 13 and Figure 14 show that the binder width and warp (weft) width have great influence on the equivalent tensile stiffness $A_{11}$ and bending stiffness $D_{11}$. The values of $A_{11}$ and $D_{11}$ increase with the increasing warp (weft) width, but decrease with the increasing binder width. The main reason is that the increase of binder width will lead to the decrease of yarn content, further resulting in the decrease of $A_{11}$ and $D_{11}$. However, the smaller the binder yarn width is, the smaller the constraint in the thickness direction will be, resulting in the easy delamination. Therefore, the binder yarn width should be adjusted to ensure enough stiffness and integrity in the design of 3D-TCP.

## 5. Comparison of Effective Performance between 2D-PWL and 3D-TCP with the Same Thickness

The 3D-TCP is developed from the 2D plain-woven laminate (2D-PWL), and has better mechanical properties. To compare the effective performance of the two textile composite plate, we establish the 2D-EPM of 2D-PWL and 3D-TCP with the same plate thickness and yarn content.

**Figure 15:**
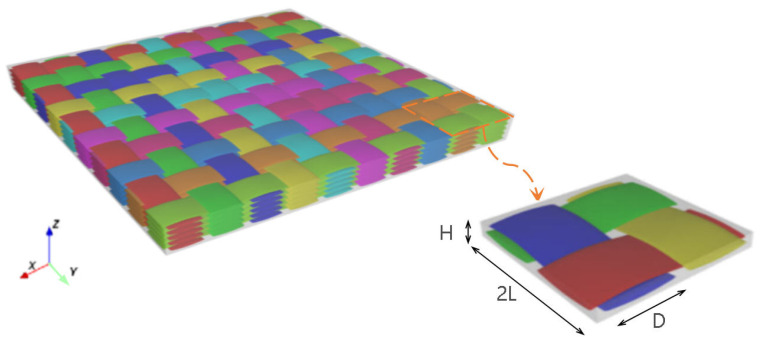
The 6-layered 2D plain-woven laminate.

The structure parameters of unit cell within the 6-layered 2D-PWL as shown in Figure 15 are: *L* = 1.2 mm, *D* = 0.8 mm, *H* = 0.2 mm. The structure parameters of unit cell within the 3D-TCP are: $b_{1}$ = 1.0 mm, $h_{1}$ = 0.2 mm, $l_{1}$ = 1.2 mm, $b_{2}$ = 0.6 mm, $h_{2}$ = 0.1 mm, $l_{2}$ = 1.2 mm, *t* = 0 mm. The thickness of both plates is 1.32 mm and the yarn content is 50.47%. The obtained equivalent stiffness matrix of 2D-PWL and 3D-TCP are shown in Table 13 and Table 14, respectively. Based on the equivalent stiffness matrix, the 2D-EPM of 150 mm × 150 mm is established to study the difference of effective performance between 2D-PWL and 3D-TCP.

### 5.1. Comparison of Bending Behaviors

**Figure 16:**
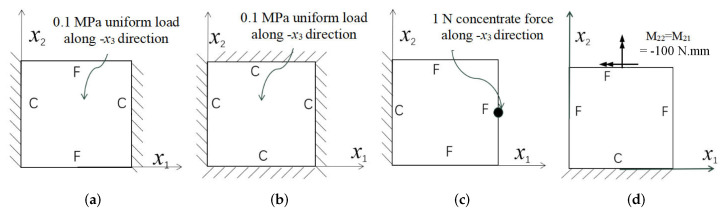
Boundary and load conditions for bending behavior analysis of 2D-PWL and 3D-TCP. (**a**) Case 8; (**b**) Case 9; (**c**) Case 10; (**d**) Case 11.

The boundary conditions used in bending analysis are shown in Figure 16, and the predicted bending displacements are shown in Table 15. It can be observed that the bending displacement of 3D-TCP is greater than that of 2D-PWL under uniform load (Case 8 and Case 9). This may due to the fact that the warp yarns and weft yarns of 3D-TCP are not interlaced, and are only constrained by the binding yarns in the thickness direction. While the displacement of 3D-TCP is smaller than that of 2D-PWL under the concentrated force and bending moment (Case 10 and Case 11), indicating that the torsion resistance of 3D-TCP are better than 2D-PWL. The above results are consistent with the obtained equivalent stiffness in Table 13 and Table 14. That is, the bending stiffness $D_{11}$ and $D_{22}$ of 2D-PWL are greater than those of 3D-TCP, while the torsional stiffness $D_{66}$ of 2D-PWL is smaller than that of 3D-TCP.

### 5.2. Comparison of Buckling Modes

Table 16 shows the first four buckling modes predicted by 2D-PWL and 3D-TCP under the conditions in Case 7. It can be observed that the buckling modes predicted by 2D-PWL and 3D-TCP are almost the same. That is, the first and third buckling modes, respectively, have one and two half-waves along the *Y* direction, and the second buckling mode has two half-waves along the *X* direction, the fourth buckling mode has two asymmetric half-waves along the *X* and *Y* directions. It is worth noting that the first four critical loads of 3D-TCP are greater than 2D-PWL, indicating that 3D-TCP has better stability under lateral load.

### 5.3. Comparison of Free-Vibration Characteristics

The first vibration mode and natural frequency of 2D-PWL and 3D-TCP under different boundary conditions in Figure 12 are compared as shown in Table 17. The vibration modes of 3D-TCP are consistent with those of 2D-PWL, while the natural frequency of 3D-TCP is smaller than that of 2D-PWL, which is consistent with the obtained equivalent stiffness.

## 6. Conclusions

The VAM-based equivalent model (2D-EPM) of 3D textile composite plate is established for bending, buckling and free-vibration analysis. The following conclusions can be obtained:

(1) The maximum errors of bending displacement and buckling load between 3D-FEM and 2D-EPM are within the range of engineering accuracy, and the displacement distributions along the analysis path predicted by two models have the same trend with small differences. Furthermore, the local stress, strain and displacement distributions within the recovered unit cell are well captured. In addition, the computational efficiency of 2D-EPM is greatly improved by reducing the number of nodes and elements.

(2) The width of binder/warp yarn mainly affect the stiffness components $A_{11}$ and $D_{11}$. That is, $A_{11}$ and $D_{11}$ increase with the increasing width of warp or weft yarn, while decrease with increasing width of binder yarn, which may due to the fact that the increasing width of binder yarn will decrease the yarn content.

(3) Compared with 2D plain-woven laminate with the same thickness and yarn content, the 3D textile composite plate has smaller equivalent bending stiffness and larger torsional stiffness, resulting in large displacement and small natural frequency. This may be because the 3D textile composite plate is only constrained by the binding yarns in the thickness direction, while the warp yarns and weft yarns are intertwined closely in the 2D-PWL.

## Figures and Tables

**Table 1:** Constituent properties of matrix and fiber used in the yarn [35].

Constituent Proprties	Epoxy Resin-3601	Carbon FibreT-300
Elastic modulus $E_{1}$/GPa	4.51	208.8
Elastic modulus $E_{2}$ = $E_{3}$/GPa	4.51	43
Shear modulus $G_{12}$ = $G_{13}$/GPa	1.7	7.42
Shear modulus $G_{23}$/GPa	1.7	7.42
Poisson’s ratio $v_{12}$ = $v_{13}$	0.38	0.2
Poisson’s ratio $v_{23}$	0.38	0.5

**Table 2:** Equivalent engineering constants of the yarn with a fiber volume fraction of 64%.

Equivalent Engineering Constants	Values
Elastic modulus $E_{1}$/GPa	135.28
Elastic modulus $E_{2}$ = $E_{3}$/GPa	15.21
Shear modulus $G_{12}$ = $G_{13}$/GPa	3.99
Shear modulus $G_{23}$/GPa	3.97
Poisson’s ratio $v_{12}$ = $v_{13}$	0.26
Poisson’s ratio $v_{23}$	0.51

**Table 3:** Equivalent stiffness matrix of 3D-TCP (unit: SI).

Equivalent Stiffness Matrix of 3D-TCP					
31,093.30	2999.61	0	15,546.70	1499.81	0
2999.61	10,674.90	0	1499.81	5337.46	0
0	0	2944.26	0	0	1472.13
15,546.70	1499.81	0	10,415.50	1162.04	0
1499.81	5337.46	0	1162.04	4072.26	0
0	0	1472.13	0	0	1130.14

**Table 4:** Comparison of displacement $U_{3}$ predicted by two models under various boundary and load conditions (unit: mm).

Case	3D-FEM	2D-EPM	Max. Error
Case 1	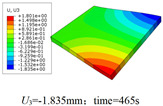	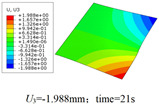	7.70%
Case 2	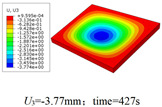	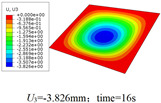	1.36%
Case 3	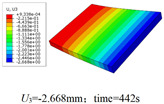	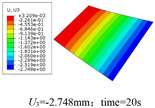	2.91%

**Table 5:** Comparison of local displacement field within the unit cell under the conditions in Case 2 (unit: mm).

Displacement	Selected Unit Cell	Recovered Unit Cell	Max. Error
*U*	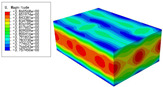	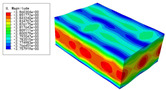	0.35%
$U_{2}$	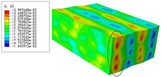	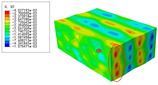	1.18%
$U_{3}$	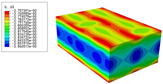	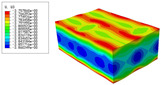	0.12%

**Table 6:** Comparison of local stress field within the unit cell under the conditions in Case 2 (unit: MPa).

Stress	Selected Unit Cell	Recovered Unit Cell	Max. Error
Von Mises	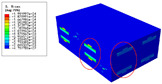	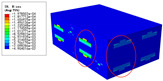	0.70%
$\sigma_{22}$	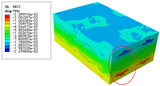	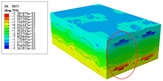	0.85%
$\sigma_{12}$	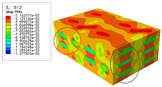	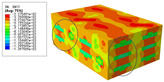	5.17%

**Table 7:** Comparison of local strain field within the unit cell under the conditions in Case 2.

Stress	Selected Unit Cell	Recovered Unit Cell	Max. Error
$\varepsilon_{11}$	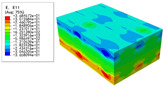	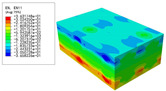	0.92%
$\varepsilon_{13}$	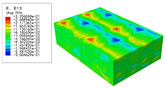	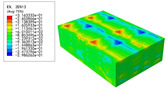	0.91%
$\varepsilon_{33}$	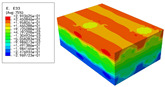	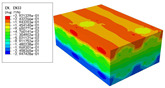	0.75%

**Table 8:** Comparison of the buckling modes and critical loads (N) between 3D-FEM and 2D-EPM under the conditions in Case 6.

Order	3D-FEM	2D-EPM	Max. Error
1	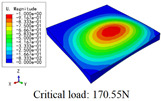	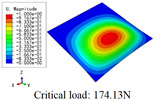	2.06%
2	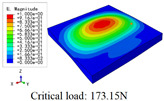	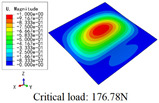	2.10%
3	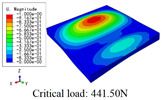	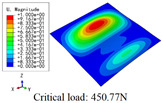	2.18%
4	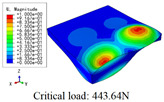	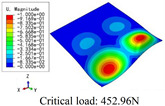	2.06%
5	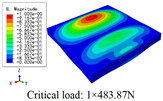	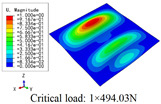	2.10%
6	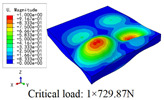	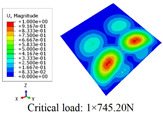	2.06%

**Table 9:** Comparison of the first buckling modes and critical loads (N) of 3D-TCP in different cases predicted by two models.

Case	3D-FEM	2D-EPM	Max. Error
Case 4	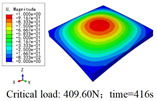	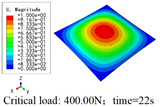	2.40%
Case 5	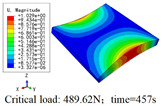	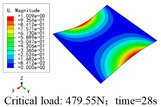	2.09%
Case 7	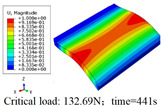	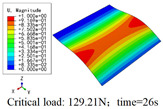	2.69%

**Table 10:** Comparison of the first three free vibration characteristics predicted by two models under the boundary condition in Case 4.

Order	3D-FEM	2D-EPM	Max. Error
1	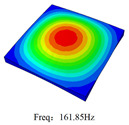	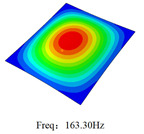	0.90%
2	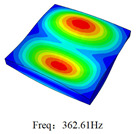	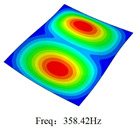	1.17%
3	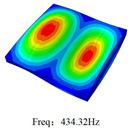	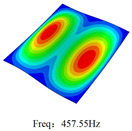	5.08%

**Table 11:** Comparison of the first four free vibration characteristics predicted by two models under the boundary condition in Case 7.

Order	3D-FEM	2D-EPM	Max. Error
1	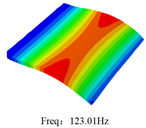	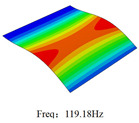	3.21%
2	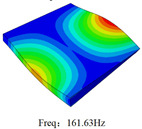	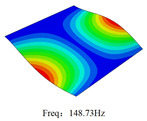	8.67%
3	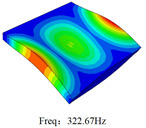	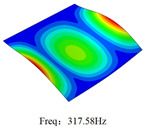	1.58%

**Table 12:** Structural parameters of 3D-TCP used in the parameter analysis.

Warp or Weft Yarn Parameters			Binder Yarn Parameters		
$b_{1}$	$h_{1}$	$l_{1}$	$b_{2}$	$h_{2}$	$l_{2}$
0.7∼1.1 mm	0.2 mm	1.2 mm	n×1.0 mm ^1^	0.1 mm	n×1.2 mm

^1^*n* is the multiplier, and can be chosen as eight different values: 0.2, 0.3, 0.4, 0.5, 0.6, 0.7, 0.8 and 0.9, respectively.

**Table 13:** Equivalent stiffness matrix of 2D-PWL (unit: SI).

Equivalent Stiffness Matrix of 2D-PWL					
58,549.00	3208.21	0.00	35,129.40	1924.92	0.00
3208.21	12,037.40	0.00	1924.92	7222.47	0.00
0.00	0.00	3219.44	0.00	0.00	1931.66
35,129.40	1924.92	0.00	27,994.70	1541.54	0.00
1924.92	7222.47	0.00	1541.54	5787.12	0.00
0.00	0.00	1931.66	0.00	0.00	1555.76

**Table 14:** Equivalent stiffness matrix of 3D-TCP (unit: SI).

Equivalent Stiffness Matrix of 3D-TCP					
45,677.30	15,832.30	0.00	0.00	0.00	−1884.16
15,832.30	17,592.60	0.00	0.00	0.00	−548.05
0.00	0.00	15,615.30	−1884.16	−548.05	0.00
0.00	0.00	−1884.16	9744.50	3377.56	0.00
0.00	0.00	−548.05	3377.56	3753.08	0.00
−1884.16	−548.05	0.00	0.00	0.00	3331.27

**Table 15:** Comparison of bending displacement between 3D-TCP and 2D-PWL under different conditions.

Cases	2D-PWL	3D-TCP
Case 8	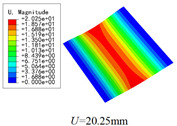	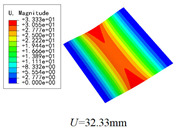
Case 9	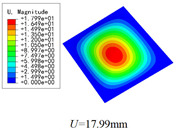	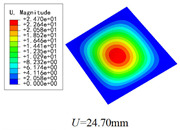
Case 10	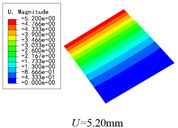	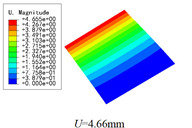
Case 11	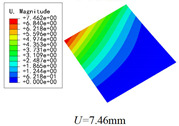	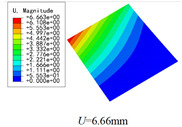

**Table 16:** Comparison of the first four buckling modes between 3D-TCP and 2D-PWL under the conditions in Case 7.

Order	2D-PWL	3D-TCP
1	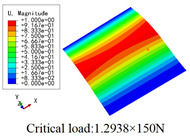	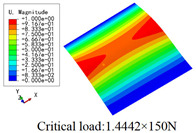
2	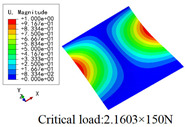	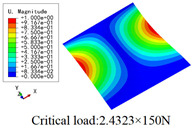
3	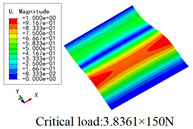	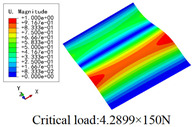
4	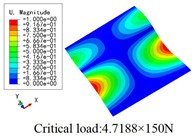	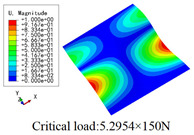

**Table 17:** Comparison of the first vibration modes and natural frequencies (Hz) predicted by 2D-PWL and 3D-TCP under different boundary conditions.

Case	2D-PWL	3D-TCP
Case 4	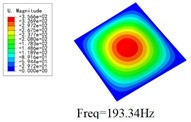	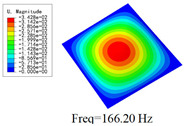
Case 6	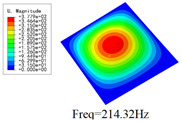	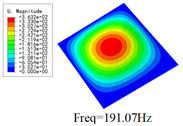
Case 7	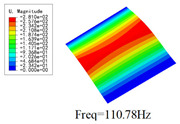	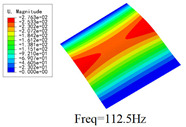

## Data Availability

Data available on request due to restrictions, e.g., privacy or ethical. The data presented in this study are available on request from the corresponding author. The data are not publicly available due to subsequent analyzes and publications.
